# The Role of the Rhizosphere, Endophytes, and the Influence of Plant-Growth-Promoting Bacteria: Take the Cannabis Microbiome as an Example

**DOI:** 10.3390/ijms27114802

**Published:** 2026-05-26

**Authors:** Piotr Stanisław Wiszpolski, Mariusz Jerzy Stolarski

**Affiliations:** 1 Department of Plant Breeding and Bioresource Engineering, Faculty of Agriculture and Forestry, University of Warmia and Mazury in Olsztyn, Plac Łódzki 3, 10-724 Olsztyn, Poland; 2 Chemprof Doradztwo Chemiczne s.c. Katarzyna Łuczyńska i Michał Łuczyński, Gutkowo 54B, 11-041 Olsztyn, Poland; 3 Centre for Bioeconomy and Renewable Energies, University of Warmia and Mazury in Olsztyn, Plac Łódzki 3, 10-724 Olsztyn, Poland

**Keywords:** *Cannabis sativa*, rhizosphere microbiota, plant growth-promoting bacteria (PGPB), phytocannabinoids, plant–microbe interactions

## Abstract

*Cannabis sativa* L. is a multipurpose crop of increasing agricultural and medical relevance, whose productivity and phytocannabinoid profile are influenced not only by genotype and environmental factors but also by the composition of its microbiota. This review synthesizes current knowledge (2020–2026) on the rhizosphere and endophytic microbiota of hemp, with particular emphasis on plant growth-promoting bacteria (PGPB) and their mechanisms of action. Molecular studies indicate that hemp-associated bacterial communities are dominated by Proteobacteria, Actinobacteriota, *Firmicutes* and Bacteroidota, with genotype-, tissue- and developmental-stage-dependent variation. PGPB influence plant performance through direct mechanisms, including biological nitrogen fixation, phosphate solubilization, siderophore production and phytohormone synthesis (indole-3-acetic acid (IAA), gibberellins, cytokinins, and 1-aminocyclopropane-1-carboxylate (ACC) deaminase), as well as indirect mechanisms such as antibiosis, enzyme-mediated pathogen inhibition and induction of systemic tolerance to abiotic stress. Experimental studies demonstrate that inoculation with selected strains or consortia can enhance biomass accumulation, improve germination and root architecture, increase resistance to *Fusarium oxysporum* and modulate cannabinoid and terpene profiles. Importantly, plant responses are cultivar-specific, highlighting the need for genotype-tailored microbial formulations.

## 1. Introduction

### 1.1. Biology and Economic Importance of Cannabis Sativa

*Cannabis sativa* ssp. is an annual herbaceous flowering plant. It originated in Asia, but it is cultivated worldwide today. Hemp has been known since ancient times; there is evidence that the ancient Chinese used it as a food, a source of fiber and for ritual rites [1,2,3]. Hemp fiber was also used in medieval Europe as a fiber material and for rope production [4,5]. It was also used in traditional medicine [6]. The invention of synthetic fibers and the perception of hemp as a drug almost stopped the cultivation of this plant in Europe. The maximum permitted THC content in industrial hemp in the European Union was increased from 0.2% to 0.3% under the Common Agricultural Policy (CAP) reform adopted in 2021, with the new threshold applied from 1 January 2023 [7]. Hemp farmers must exclusively use certified seed from varieties listed in the EU Common Catalog of Varieties of Agricultural Plant Species. Nowadays, hemp is a multipurpose raw material for food, feed, cosmetics, dietary supplements, medicines, textiles, building materials, bioplastic production, and the production of energetic materials [8,9].

Cannabis is rich in phytochemical compounds, such as cannabinoids, flavonoids, and sesquiterpenes [10]. The most important group are cannabinoids. The group contains over 100 active chemical compounds but the primary ones are cannabidiol (CBD), cannabigerol (CBG) and tetrahydrocannabinol (THC) [11,12,13]. CBD is particularly important due to its low psychoactivity and beneficial therapeutic effects in the treatment of diseases and disorders such as neurodegenerative diseases, rheumatoid arthritis, neurological disorders, depression and epilepsy [6,13,14].

Hemp is adapted to grow in various climatic conditions in many regions of the world [15]. It is characterized by a short growing season, high biomass yield, and drought resistance and does not require the use of large amounts of insecticides [16]. Thanks to its rapid growth, hemp creates shade zones, limiting weed growth. These characteristics make hemp a valuable plant in intercropping strategies, reducing the risk of weed and crop pest spread and allowing for reduced pesticide and herbicide use. *Cannabis sativa* also bioaccumulates heavy metals and can be successfully used in soil phytoremediation [12,15].

### 1.2. Microbial Strategies for Sustainable Hemp Cultivation

Scientific progress is increasing awareness of the impact of agricultural practices, particularly fertilization, herbicides and pesticides, on the natural environment and consumer health. Plant protection products are particularly dangerous, negatively impacting fertility, carcinogenesis and the central nervous system [17]. Chemical residues also end up in groundwater, degrading its quality. Chemical fertilizers, along with surface runoff, reach water bodies, enriching the water with the nutrient’s phosphorus and nitrogen, which in turn leads to the rapid growth of aquatic algae [18,19,20]. Eutrophication of water possessions is a threat to human and animal life and also causes economic losses and leads to irreversible ecological changes.

The impacts of chemical intensification on human and animal health and ecosystems have been increasingly documented, leading to actions aimed at environmental protection [17,20,21,22]. The European Union’s Green Deal policy not only aims to reduce carbon dioxide emissions but also sets goals for agriculture, such as reducing the use of chemical pesticides by 50% and limiting the use of fertilizers. The Farm to Fork Strategy also emphasizes limiting the use of antimicrobials in animal husbandry and aquaculture and implementing organic production on 25% of the EU’s agricultural land. Climate change, scientific advances and growing consumer awareness are impacting agriculture and exerting pressure for change. Hemp is a plant with wide applications in many industries, characterized by rapid growth, resistance to drought, weeds and pests and a high capacity for carbon sequestration [23].

Soil conditions and fertility are closely linked to its microbial composition. The consortium of microorganisms is responsible for nutrient cycling and the synthesis and transformation of chemical compounds. The microbiology of the rhizosphere is particularly important because it determines the interactions between microorganisms and plants. Currently, isolates of beneficial microorganisms are used to create microbiological preparations that support plant growth and biosecurity. This is particularly important in terms of reducing the use of chemicals in agriculture, food safety and protecting biodiversity. The high potential of microorganisms to influence cannabis encourages further research; however, studies evaluating the effectiveness of microbiological preparations under field cultivation conditions are essential.

The aim of this work is to collect scientific reports on the microbiota of hemp (*Cannabis sativa* ssp.), its effect on growth, characteristics of secondary metabolite production and the use of growth-promoting bacteria.

## 2. Results and Discussion

### 2.1. The Mechanism of Action of Soil Bacteria on Plants

**Figure 1:**
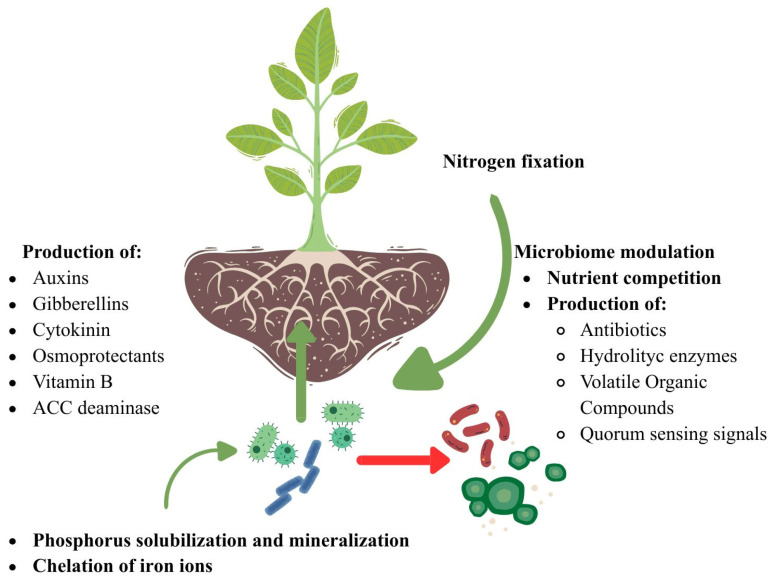
The interactions between plant and rhizosphere microbiota.

The impact of microorganisms on plants can be divided into direct and indirect actions. Indirect action involves regulating competition, producing antibiotics, hydrogen cyanide, or enzymes that degrade the cell walls of pathogenic fungi [22,24,25,26]. Direct action involves the production of siderophores, phytohormones such as indole-3-acetic acid, gibberellins and cytokinins, as well as nitrogen fixation and phosphorus solubilization [24,25,26,27]. Additionally, in response to environmental stress, microorganisms can produce compounds that prevent drought or frost. These actions are associated with both symbiotic plant bacteria and free-living soil bacteria associated with plant tissues (endophytes), as well as organisms living in the rhizosphere (Figure 1).

#### 2.1.1. Atmospheric Nitrogen Fixation

Nitrogen, along with carbon and phosphorus, is the main nutrient. It is also the main component of the Earth’s atmosphere, accounting for 78%, but plants cannot bind it [26]. Some soil bacteria can process atmospheric nitrogen into forms available to plants [28,29,30]. These microorganisms occur as plant symbionts, e.g., *Rhizobium* spp. with legumes, or as free-living bacteria, e.g., *Azotobacter* spp., *Azospirillum* spp., *Pseudomonas* spp., and *Bacillus* spp. [30,31].

Nitrogen fixation is possible thanks to the action of the nitrogenase enzyme. This mechanism enables bacterial growth in nitrogen-poor environments. The nif genes encode the ability to produce the enzyme and its activity is conditioned by the presence of trace elements such as iron and molybdenum ions, anaerobic conditions, appropriate pH and energy [28,29]. The nitrogenase complex is highly sensitive to oxygen and nitrogen-fixing bacteria have defense strategies against excessive oxygen concentrations, such as the production of polymers or oxygen-scavenging proteins and the formation of biofilms [32]. The process itself is energy-intensive; the reduction of one nitrogen molecule requires the hydrolysis of 16 adenosine triphosphate (ATP) molecules [28,32].

Cannabis does not form a symbiotic relationship with nitrogen-fixing bacteria, but free-living microorganisms can have a positive impact on plant growth. A consortium of microorganisms has been shown to positively impact cannabis morphological characteristics as well as Soil-Plant Analysis Development (SPAD) values, which correlate with N content [33].

#### 2.1.2. Phosphorus Solubilization

Phosphorus is an essential element, a component of basic chemical compounds such as deoxyribonucleic acid (DNA), ribonucleic acid (RNA), proteins and the energy carrier ATP. By regulating phosphorus metabolism, plants can respond to stress conditions. It occurs naturally in organic and mineral forms. Plants can take up soluble forms of phosphorus, such as orthophosphates. Organic forms, such as phytates, must first be mineralized, while mineral forms, such as apatite and calcium phosphate, must be solubilized. Soil pH is crucial; plant-available forms are present at pH 6.5–7.2. In alkaline soils, insoluble calcium and magnesium salts predominate, while, in acidic soils, iron and aluminum salts are inaccessible [34]. Fertilizers based on superphosphates and ammonium phosphates are used in agriculture. However, it is estimated that only 10–25% is absorbed by plants; the rest is washed away, causing soil contamination and water eutrophication [35].

The solubilization process is carried out by several soil microorganisms, including those from the following genera: *Bacillus* spp., *Pseudomonas* spp., *Streptomyces* spp., *Aspergillus* spp., *Rhizobium* spp., *Fusarium* spp., *Trichoderma* spp., *Penicillium* spp., *Seratia* spp., *Acinetobacter* spp., *Agrobacterium* spp. *Arthrobacter* spp., *Burkholderia* spp., and *Rahnella* spp. [34,36]. Several mechanisms are responsible for the ability of microorganisms to solubilize insoluble forms of phosphorus, the most important of which are the production of organic acids, the production of H_2_S, exopolysaccharides and siderophores [35]. The main mechanism is the production of organic acids such as gluconic acid, lactic acid, acetic acid, propionic acid, formic acid, malic acid and succinic acid [26,36,37,38]. These compounds chelate iron, calcium and aluminum ions in the soil, releasing phosphates into the soil [38]. Another mechanism involves the production of H_2_S by acidophilic sulfur-oxidizing bacteria, which can react with iron phosphate, producing iron sulfide and a phosphate group. Bacteria can also release protons into the environment, particularly when the nitrogen source is ammonium salts. During the conversion of NH_4_^+^ to NH_3_, H^+^ is released, leading to environmental acidification, which in turn affects the solubilization of insoluble phosphates. Another mechanism involves the production of exopolysaccharides, polymers composed of carbohydrate groups secreted outside the cell. These compounds participate in biofilm formation and can form complexes with soil metal ions, releasing their associated phosphorus reserves. Siderophores are low-molecular-weight compounds that strongly chelate iron, produced by both plants and bacteria. Binding iron ions allows plants to access phosphorus stored in the soil.

Phosphorus availability affects plant growth and development, including yield and chemical composition [39]. Studies by Contant et al. [40] demonstrated a positive effect of the Mammoth PTM bacterial consortium on bud yield and height of cannabis, while Vadhel et al. [41] demonstrated a positive effect of solubilizing bacteria on root and shoot length and cannabinoid content.

#### 2.1.3. Production of Phytohormones

Soil bacteria can produce a range of phytohormones. Their importance is greatest in stressful situations such as drought, nutrient deprivation, salinity, or the presence of heavy metal ions. Microorganisms can synthesize auxins, cytokinins, gibberellins and 1-aminocyclopropane-1-carboxylate (ACC) deaminase [30,32,42].

The best-known phytohormone produced by soil microorganisms is indole-3-acetic acid. It promotes cell division and growth, as well as rooting and fruiting. It is estimated that 80% of microorganisms in the root zone can synthesize this compound. These include bacteria of the following genera: *Acetobacter* spp., *Acinetobacter* spp., *Azospirillum* spp., *Arthrobacter* spp., *Azotobacter* spp., *Bacillus* spp., *Burkholderia* spp., *Peanibacillus* spp., *Pseudomonas* spp., *Rhizobium* spp., *Rhodococcus* spp., *Serratia* spp., and *Streptomyces* spp. [43]. The hormone’s precursor is L-tryptophan [26,34,35,44,45].

Gibberellins, particularly gibberellic acid, are responsible for seed germination, shoot elongation, flowering and fruit set in plants [34]. These phytohormones include over 130 chemical compounds with diverse activities. There is evidence of gibberellin production by bacteria from the genera *Achromobacter* spp., *Gluconobacter* spp., *Acinetobacter* spp., *Bacillus* spp., *Rhizobium* spp., *Azotobacter* spp. and *Azospirillum* spp. [43].

Cytokinins are phytohormones whose precursor is adenine. They act in synergy with auxins, maintaining the balance between root and shoot growth, influencing cell division, organ formation and seed germination. They regulate nutrient transport and chlorophyll biosynthesis. Bacteria from the *Agrobacterium* spp., *Bacillus* spp., *Methylobacterium* spp., *Bradhyrizobium* spp. and *Pseudomonas* spp. groups can synthesize these phytohormones and are associated with the presence of the *ipt* gene, encoding the enzyme isopentenyltransferase (IPT) [43].

Ethylene is a gaseous plant hormone involved in regulating growth, development and plant stress responses. It plays an important role in fruit ripening, leaf shedding and response to drought or damage [26,43]. However, high ethylene concentrations can inhibit growth. The precursor of ethylene in plant cells is ACC—1-aminocyclopropane-1-carboxylic acid, which is converted to ethylene by ACC oxidase. Selected strains of soil bacteria possess the enzyme ACC deaminase, which degrades the precursor into alpha-ketobutyrate and ammonia, thereby reducing the potential for stress-causing ethylene formation while simultaneously providing the plant with nitrogen. This enzyme is present in selected strains of bacteria from the following genera: *Pseudomonas* spp., *Serratia* spp., *Staphylococcus* spp., *Burkholderia* spp., *Bacillus* spp. and *Agrobacterium* spp. [26,43].

#### 2.1.4. Drought Prevention Measures

Prolonged drought causes a range of physiological, biochemical and molecular changes in plants. It inhibits plant growth and development, photosynthesis, phytohormone production and the transport of nutrients, micronutrients and macronutrients. Plants have their own mechanisms of response to drought: they close stomata, slow down photosynthesis, accumulate osmotic substances such as proline, glycine or betaine to retain water in the cell cytoplasm and secrete antioxidant substances to prevent the negative effects of free radicals [46].

Microorganisms possess several abilities that help mitigate the effects of drought. These mechanisms include the production of exopolysaccharides, which increase water retention and form a water-retaining coating around the root [46,47]. They also produce compounds with antioxidant properties, including enzymes peroxidase and catalase, which scavenge free radicals and prevent cell membrane degradation [30]. Their ability to produce phytohormones (indole-3-acetic acid), which helps plants develop strong root systems, is also important, as is ACC deaminase, which reduces the effects of ethylene [30,48]. Microorganisms can also produce osmoregulatory compounds, such as proline and trehalose, which help maintain water turgor and osmotic balance [46,49]. At the molecular level, bacteria activate plant genes responsible for drought response, such as dehydration-responsive element-binding (*DREB*), responsive to desiccation 29A (*RD29A*) and late embryogenesis abundant (*LEA*), which encode cell-stabilizing and osmoprotective proteins, as well as genes encoding antioxidant enzymes, including superoxide dismutase (SOD), catalase (CAT) and ascorbate peroxidase (APX) [46,50]. Microorganisms involved in the drought response include *Bacillus* spp., *Pseudomonas* spp., *Rhizobium* spp., *Azospirillum* spp. and *Enterobacter* spp.

#### 2.1.5. Competition

Plants actively control the composition of the root zone microbiota through the substances they secrete, including attractants from selected groups. These microorganisms can inhibit microbial growth. Bacteria produce a range of chemical compounds, such as chitinases, cellulases, beta-1,3-glucanases, proteases and lipases, which can cause damage to cell walls and lead to cell lysis [50,51]. Bacteria of the *Pseudomonas* genus are capable of producing antibiotics, such as 2,4-diacetylphloroglucinol or phenazine-1-carboxylate, which inhibit fungal growth [44]. Selected strains of *Bacillus* spp. bacteria produce surfactants, such as surfactin or iturins, which affect the cell membranes of bacteria and fungi [37,45,52]. Bacteriocins, unlike antibiotics, which act on a narrower spectrum, are also important in competition for lower ecological status. Siderophores also play an indirect role, limiting the availability of iron to potentially pathogenic organisms [42,53,54]. For plant growth-promoting bacteria, antagonistic effects have been reported against fungi such as Botrytis spp., Fusarium spp., Sclerotium spp. and Phytophthora spp. [55].

### 2.2. The Effect of Bacteria on Cannabis Sativa

#### 2.2.1. Culture-Based Studies on Bacterial Effects on *Cannabis sativa* spp.

Between 2020 and 2026, the impact of microorganisms and their consortia on *Cannabis* spp. was repeatedly analyzed. Studies considered plant growth parameters such as height, dry and fresh tissue weight, as well as the impact on germination and chemical parameters, including THC and cannabinoid content, polyphenol content and antioxidant properties. These studies used bacterial isolates with previously documented growth-promoting effects, isolates from hemp seeds or tissues, or commercial preparations (Table 1).

An important mechanism that increases the effectiveness of inoculation is the interaction among various microorganisms. Comeau et al. [56] analyzed the effect of bacteria from the genera *Bacillus* (*B. subtilis* LBUM979, *B. siamensis* LBUM1082, and *B. velezensis* LBUM279) and *Pseudomonas* (*P. fluorescens* LBUM223 and *P. protegens* LBUM825) isolated from the root zone of *Fragaria ananassa*. A pot experiment conducted on the “Anka” cannabis cultivar showed that single strains did not significantly affect the dry weight of aboveground and underground plant parts, whereas bacterial combinations significantly increased biomass. In Promix medium, plant weight increased by approximately 30% for the LBUM223/979, LBUM223/1082 and LBUM825/979 pairs. A similar effect was observed in Canna Coco medium. Importantly, a double dose of a single Pseudomonas strain did not cause significant changes, whereas a high dose of *Bacillus* negatively affected plant vigor. These results indicate that microbial synergy, not just their concentration, is crucial. A similar approach was used in aquaponic systems. Marin-Campos et al. [67] studied a formulation containing *Pseudomonas fluorescens*, *Trichoderma harzianum*, *T. viride* and *T. reesei*. They demonstrated that the optimal inoculant dose (6.67 × 10^4^ CFU/L) increased plant height, stem and root diameter and leaf and stem biomass. This increase in biomass was also accompanied by an increase in flavonoid content. The authors attribute these effects to the production of growth regulators, siderophores and hydrolytic enzymes that increase the availability of mineral nutrients.

One of the most frequently described mechanisms of microbial action is improving nutrient availability through mineral solubilization, metal ion chelating and increasing the absorptive surface area of the root system. Research by Kakabouki et al. [57,58] showed that inoculation of hemp with preparations containing *Rhizopus irregularis* and *Trichoderma harzianum* led to increased root length, plant height and dry biomass. These effects were attributed to the microorganisms’ ability to solubilize elements such as Fe, Mn, Zn and P and to produce phytohormones, including indole-3-acetic acid and gibberellins. The extensive mycelial network also increased the absorptive surface area of the root system, improving plant water management. A similar phenomenon was observed with mycorrhizal fungi of the genus *Rhizophagus*. Seemakram et al. [61] demonstrated that inoculation with the *Rhizophagus aggregatus* BM-3 g3 strain significantly increased morphometric parameters of cannabis, including plant height, leaf area and dry weight of stems, leaves and inflorescences. Inoculation also influenced secondary metabolism, increasing CBD and THC content.

In addition to influencing plant growth, microorganisms can modulate the biosynthesis of secondary metabolites, including cannabinoids and terpenes. Ahmed et al. [64] demonstrated that the use of Ferticann, containing *Rhizopus irregularis*, *Trichoderma harzianum* and *Bacillus subtilis*, among others, resulted in changes in the cannabinoid composition of various medical cannabis varieties. Depending on the variety, changes in the content of cannabidivarin (CBDV), cannabigerol (CBG), cannabigerolic acid (CBGA) and tetrahydrocannabinolic acid (THCA) were observed. Similar observations were made by Lyu et al. [63], who studied the effects of bacteria from the genera *Bacillus*, *Pseudomonas* and *Mucilaginibacter*. Inoculation increased plant biomass and the number of flower buds and influenced the secondary metabolite profile. *Mucilaginibacter* increased cannabinoid and terpene content, while *Pseudomonas* primarily affected inflorescence yield. The influence of microorganisms on secondary metabolism has also been demonstrated in aquaponic systems. Tonolo et al. [69] found that inoculation with bacteria from the genera *Bacillus* spp., *Pseudomonas* spp., *Flavobacterium* spp. and *Burkholderia* spp. did not significantly affect plant morphology but increased CBGA content and reduced THCA.

Seed-associated endophytes are a significant source of microorganisms supporting hemp development. Gabriel et al. [60] isolated 36 bacteria from seeds and seedlings of the Futura 75 cultivar. One strain, belonging to the genus *Sphingomonas*, did not significantly affect seedling morphometric parameters but increased the content of polyphenols and compounds with antioxidant activity. Studies on seed microbiota also indicate the potential of bacteria from the genus *Bacillus*. Lobato et al. [65] demonstrated that the endophytic strain *Bacillus frigoritolerans* increased the yield of the fiber hemp cultivar Eletta Campana under field conditions by up to threefold compared to the control.

Rhizosphere microorganisms can also limit the development of plant pathogens by producing antifungal metabolites. Pellegrini et al. [59] demonstrated that a consortium of *Azospirillum brasilense*, *Gluconacetobacter diazotrophicus*, *Herbaspirillum seropedicae* and *Burkholderia ambifaria* inhibited the growth of *Fusarium oxysporum* by approximately 70% in in vitro assays. In pot experiments, the consortium increased the survival of infected plants by up to 85%, compared to 42% in the control group. This mechanism is associated with the production of antifungal metabolites such as pyrrolnitrin and burkholdins, as well as volatile sulfur compounds. Similar properties are observed in bacteria of the genus *Bacillus*, which produce a wide range of bioactive compounds, including siderophores, bacteriocins, lytic enzymes and volatile organic compounds. Corredor-Perilla et al. [62] demonstrated that *Bacillus* spp. isolates from the hemp rhizosphere could simultaneously stimulate seed germination and inhibit the development of *Fusarium oxysporum*. Some strains increased germination efficiency from 60% in the control group to 100%.

Bacteria of the genera *Bacillus* and *Pseudomonas* are characterized by their ability to colonize the root system rapidly and, in some cases, other plant tissues. Aunkam et al. [66] demonstrated that the *Bacillus velezensis* S141 strain colonized not only cannabis roots but also stems and leaves. Inoculation increased plant dry weight and chlorophyll content.

Research on the Cannabis rhizosphere microbiome also indicates the potential of other plant growth-promoting bacteria, such as *Burkholderia cepacia* or *Rhizobium scorae*, which increased plant biomass by up to 62–64% in pot experiments [68].

#### 2.2.2. Microbiota Studies Using Molecular Methods

Due to the difficulties of isolating and culturing many microorganisms under laboratory conditions, molecular methods based on DNA amplification and sequencing are now widely used to characterize the plant microbiome. These techniques also enable the identification of microorganisms that do not grow on standard microbiological media, thus enabling a much more comprehensive understanding of the microbiological structure of the studied environment. Numerous studies describing the qualitative and quantitative composition of microorganisms associated with *Cannabis sativa* spp. have been published in recent years (Table 2).

One factor significantly shaping the structure of the cannabis microbiome is the plant’s developmental stage. Research by Guo et al. [70] demonstrated significant changes in the abundance and diversity of soil bacteria during plant ontogeny. The highest abundance of microorganisms was observed at the seedling stage, then declined during the intensive growth and flowering phases, and rebounded during plant maturation. Species diversity, as measured by the Shannon index, exhibited a different dynamic, with the highest values during the seedling and flowering phases and the lowest during maturation. The authors suggest that the dominance of rapidly growing bacteria in the initial and final stages of development may be related to their ability to utilize plant debris as a nutrient source effectively. Simultaneously, the reduction in microorganism abundance during intensive plant growth may be linked to the action of compounds secreted by roots that modulate the composition of the rhizosphere microbiota [70]. Similar observations regarding microbiome variability during plant development were presented by Comeau et al. [75], who demonstrated that the Shannon index increases until the prevegetative phase, then stabilizes in subsequent growth stages. As plants develop, the share of dominant taxa also changes, with the proportion of Proteobacteria gradually decreasing in favor of Actinobacteria. The authors also indicate that microbiome variability is more strongly associated with the plant development stage than with its genotype [75].

Another important factor determining the structure of the microbiome is the type of plant tissue. Metagenomic analyses show a clear gradation in microbial diversity between individual plant organs. In a study by Willman et al. [77], covering soil, roots, leaves and flower buds of the Tangerine cultivar, the highest number of bacterial amplicon variants (bASVs) was found in soil (2170 bASVs), followed by roots (1141 bASVs) and leaves (342 bASVs), and the lowest in flower buds (181 bASVs). In above-ground tissues, Gammaproteobacteria and Alphaproteobacteria dominated, while, in roots, in addition to Gammaproteobacteria, Bacteroidia and Actinobacteria were also abundant. In soil, the dominant groups were Alphaproteobacteria, Gammaproteobacteria and Deltaproteobacteria [77]. Similar relationships were noted in a study by Greetatorn et al. [68], which analyzed the microbiome of various plant parts of the medicinal cultivar Foi Thong Suranaree 1. The highest biodiversity was found in the soil of the plant root zone grown in the field, while the lowest was found in the aboveground tissues. In the buds of field-grown plants, Gammaproteobacteria (93.02%), Alphaproteobacteria (4.08%), and Bacilli (1.91%) dominated, whereas, in greenhouse conditions, Gammaproteobacteria accounted for almost all endophytes in leaves and buds. The greatest diversity in bacterial composition was observed in the roots of plants grown in the soil, where Gammaproteobacteria (83.56%) and Alphaproteobacteria (13.35%) dominated. The composition of the root microbiome differed significantly from that of pot-grown plants, where Actinobacteria (57.92%) and Gammaproteobacteria (40.17%) dominated [68]. A similar microbiome structure was described by Barnett et al. [73], who demonstrated the dominance of Actinobacteria, Gammaproteobacteria and Betaproteobacteria in the root tissues of the Anka cannabis cultivar. At the same time, Alphaproteobacteria dominated in the leaves and Firmicutes and Gammaproteobacteria in the flowers. The authors also indicate that some microorganisms abundant in plant tissues do not dominate in soil, suggesting that plants may selectively promote specific taxa [73].

Bacterial colonization also plays a significant role in shaping the plant microbiome. Analyses conducted by Wei et al. [74] showed that the highest microbial diversity was found in the root endosphere and rhizosphere soil, while the lowest was found in above-ground tissues such as flowers, stems and leaves. Bacteria from the phyla Proteobacteria, Actinobacteria and Bacteroidetes dominated in the soil and root endosphere, while Cyanobacteria and Firmicutes were more common in flowers. Analyses of colonization sources indicated that 53.32% of the bacteria present in the rhizosphere originated directly from the soil and nearly half of the root endophytes originated from the rhizosphere microbiota. Furthermore, as many as 86.04% of the bacteria present in flowers were taxa previously present in leaves, suggesting gradual migration of microorganisms within the plant [74].

Genetic factors, such as genotype, degree of domestication and ploidy also influence plant microbiome composition. Srivastava et al. [71] demonstrated significant differences in microbial biodiversity between diploid and triploid Suver Haze plants, with Shannon–Weiner indices of 2.65 and 0.53, respectively. Reductions in microbiome diversity with increasing ploidy are also observed in other plant species. Lobato et al. [65] demonstrated that cannabis genotype accounts for 53.6% of the variability in seed bacterial microbiome composition, while degree of domestication and chemotype explain 9.66% and 6.14% of this variability, respectively. The highest microbial biodiversity was found in the least domesticated cultivars and the lowest in inbred lines. In the case of the latter, a strong simplification of the microbiome structure was observed, with *Pantoea agglomerans* being the dominant species, constituting over 80% of the seed microbiota [72]. Analysis of the core microbiome revealed several bacteria in most of the studied samples, including Pelomonas, Ralstonia, Burkholderia spp., Pseudomonas spp., Enhydrobacter, and *Rhodococcus erythropolis*.

Seed microbiome studies also revealed significant differences between the cultivated and uncultivated fractions of microorganisms. The cultivated fraction accounted for only 6.3% of all detected amplicon variants but as much as 89.2% of the total microbial population. It was dominated by rapidly growing bacteria from the genera *Pantoea*, *Bacillus* and *Pseudomonas*. The uncultivated fraction, in turn, included numerous rare taxa, often phylogenetically distant from the cultivated bacteria, which likely function in complex networks of metabolic interdependencies, making their isolation in the laboratory difficult [72].

Environmental and agrotechnical factors, including substrate type and cultivation system also influence the structure of the cannabis microbiome. Research by Comeau et al. [56] showed that commercial Canna coco and Promix substrates are dominated by bacteria belonging to the phyla Proteobacteria, Actinobacteriota, Bacteroidota, Planctomycetota, Verrucomicrobiota and Acidobacteriota. Still, their quantitative share differs between the soil and the rhizosphere. The rhizosphere showed higher alpha diversity than the non-root-associated soil. The introduction of *Bacillus* spp. primarily influenced changes in the beta diversity of the microbiome, particularly in Promix [56]. Similarly, B. Ahmed et al. [64] showed that inoculation of plants with microbiological preparations leads to changes in the structure of the rhizosphere microbiome, increasing the share of bacteria with plant growth-promoting potential, such as Streptomyces spp., *Rhizobium* spp., *Bradyrhizobium* spp., and *Mesorhizobium* spp.

Cropping systems also significantly impact the structure of the soil microbiome. Tang et al. [76] demonstrated that monoculture systems exhibit significantly lower soil microbial biodiversity than rotational systems. Crop rotation favored the development of potentially beneficial bacteria, including *Actinobacteria* spp., *Pseudomonas* spp., *Rhizobium* spp., *Flavobacterium* spp. and *Nitrospira* spp., which may play an important role in soil ecosystem functioning and plant growth [56].

#### 2.2.3. Negative Effects of Bacteria

Microorganisms isolated from the cannabis rhizosphere or other plant species, despite possessing plant growth-promoting traits, may not always exert beneficial effects and, in some cases, can negatively affect plant development. Examples include bacteria belonging to the genera *Agrobacterium* and *Pseudomonas*, which are capable of phytohormone production and phosphate solubilization, while simultaneously inducing pathological symptoms such as root gall formation and blight in hemp plants [79].

Several studies have also demonstrated that potentially beneficial bacteria can have adverse effects on seed germination and seedling development. Gabriele et al. [60] reported that inoculation with *Sphingomonas* spp. negatively affected seed germination and seedling morphometric traits, while simultaneously increasing polyphenol content and antioxidant activity. Similar observations were reported by Corredor et al. [62], where selected *Bacillus* spp. strains or bacterial consortia reduced germination rates by 30–90% and markedly inhibited seedling elongation.

The effects of bacterial inoculation are also strongly dose-dependent. Pagnani et al. [33] evaluated the influence of a bacterial consortium applied at concentrations of 10^6^ CFU/mL and 10^7^ CFU/mL. Plants inoculated with the lower dose exhibited greater stem length, dry biomass accumulation, and SPAD values than those treated with the higher concentration. Likewise, Aunkam et al. [66] demonstrated that the optimal concentration of *Bacillus velezensis* S141 was 10^6^ CFU/mL, whereas inoculation with 10^4^ CFU/mL did not significantly improve SPAD values or the dry biomass of roots, leaves, and stems. Marin-Campos et al. [67] similarly showed that inoculum concentration plays a critical role in determining plant responses. In their study, the commercial preparation “Micorrizas,” consisting of Pseudomonas fluorescens and Trichoderma spp., improved morphometric parameters at 10^4^ CFU/mL, whereas a concentration of 10^5^ CFU/mL negatively affected root length and antioxidant compound content.

Dose-dependent effects have also been observed in other plant species—for example, Tariq et al. [80] demonstrated that, among inoculation levels of 10^6^, 10^7^, and 10^8^ CFU/mL of *Bacillus* spp., the concentration of 10^7^ CFU/mL produced the greatest improvement in soybean seed germination. Importantly, only two of the three tested strains exerted significant positive effects, while one did not significantly influence germination.

#### 2.2.4. Research Gaps and Limitations

Until recently, legislative restrictions associated with hemp cultivation, particularly strict limits on tetrahydrocannabinol (THC) content, substantially limited both agricultural and scientific interest in cannabis cultivation. Industrial hemp production was associated with economic and legal risks, as environmental stressors such as drought or intense solar radiation could increase THC concentrations above the permitted threshold. This uncertainty negatively affected the attractiveness of hemp cultivation and hindered the establishment of large-scale experimental studies.

A major turning point occurred in 2018 in the United States, when hemp containing less than 0.3% THC was removed from the Controlled Substances Act. Similar regulatory changes were implemented in the European Union, where the THC threshold for industrial hemp was increased from 0.2% to 0.3% in 2020 as part of the reform of the Common Agricultural Policy (CAP). Simultaneously, the rapid expansion of the cannabidiol (CBD) market increased the industrial and commercial relevance of *Cannabis* spp., stimulating interest among both growers and researchers. These regulatory changes also facilitated access to certified plant material and simplified the approval process for experimental cultivation.

Recent studies have demonstrated that numerous interacting factors shape the cannabis microbiome. The extensive diversity of cultivars, genotypes, and chemotypes and the cosmopolitan nature of *Cannabis* spp. creates substantial knowledge gaps regarding microbiome composition across different varieties and geographic regions. Consequently, several fundamental questions remain unresolved. For example, it is still unclear whether a conserved “core microbiome” exists across genetically and chemically distinct cannabis cultivars. In addition, the mechanisms responsible for microbiome assembly remain poorly understood. The contribution of vertically transmitted microorganisms and their role in shaping root-associated and rhizosphere microbial communities has not yet been fully elucidated.

Current research has focused predominantly on seed- and rhizosphere-associated microbiomes, whereas microbial communities inhabiting inflorescences and trichomes remain poorly characterized. Their potential role in regulating secondary metabolite biosynthesis is also largely unknown. Similarly, the effects of microorganisms on cannabinoid, terpene, and polyphenol production remain insufficiently understood. Available evidence suggests strong strain- and genotype-dependent interactions, which may result in either increases or decreases in THC and CBD concentrations. An important unresolved issue is the relative contribution of environmental stress and microbial activity to these metabolic shifts, as well as the extent to which these factors interact synergistically.

**Figure 2:**
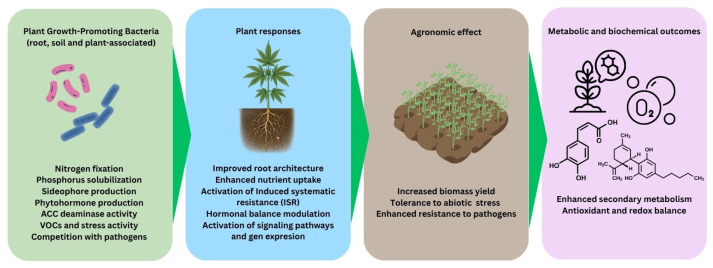
Supposed regulatory path of PGPB on *Cannabis* spp.

The mechanisms through which bacteria positively influence plant growth and development are relatively well characterized in cereals and other economically important crops. Processes such as phytohormone production, induced systemic resistance (ISR), and enhanced nutrient availability have been described not only at the phenotypic level but also in considerable molecular detail. Although several studies have reported beneficial effects of microorganisms on plant growth and secondary metabolite production in *Cannabis sativa*, many proposed mechanisms remain hypothetical. In particular, processes such as ISR induction or microbial modulation of pathways involved in THC and CBD biosynthesis still require validation at the molecular level. A proposed schematic model of microbial interactions affecting cannabis growth and metabolism is presented in Figure 2.

Current studies are focused primarily on metagenomic analyses, which enable taxonomic characterization of the cannabis-associated microbiome. However, understanding the mechanisms underlying plant–microorganism interactions requires integrating comprehensive multi-omics approaches. Metabolomic analyses are necessary to identify the chemical compounds involved in bidirectional plant–microbe interactions, whereas metatranscriptomic approaches may reveal mechanisms operating at the level of gene expression and provide insight into the activation of specific metabolic pathways. The integration of taxonomic, molecular, and chemical datasets with morphometric traits and secondary metabolite profiles could substantially improve our understanding of the relationships between the microbiome and *Cannabis* spp.

Another major research gap is the limited number of long-term field-scale studies. Current experiments are conducted under highly diverse cultivation systems, including pot, field, aquaponic, and hydroponic conditions. From both ecological and applied perspectives, field experiments most accurately reflect real agricultural environments. Such studies allow the investigation of interactions with native environmental microbiomes, including microbial competition, root-associated effects, and the influence of microorganisms on the chemical composition of *Cannabis* spp., including secondary metabolite production. However, field experiments are also associated with high levels of environmental variability. Factors such as temperature, humidity, solar radiation, and soil composition cannot be fully controlled and may substantially influence experimental outcomes. Consequently, obtaining reliable and reproducible results requires multi-location trials with sufficient biological replication. There is also a strong need for long-term, multi-season studies capable of minimizing the influence of seasonal variability. Such approaches require appropriate infrastructure and substantially increase the duration and cost of research.

An additional limitation arises from the lack of methodological standardization in studies investigating microorganisms associated with *Cannabis sativa*. Current research is based predominantly on sequencing of 16S rRNA gene regions and the fungal ITS region. However, studies differ considerably in methodologies related to sample collection, preparation procedures, seed or root surface sterilization, DNA extraction protocols, and bioinformatic pipelines. These methodological inconsistencies complicate direct comparisons among studies currently being conducted worldwide.

## 3. Search Strategy and Selection Criteria

This study included a review of papers on the *Cannabis sativa* spp. microbiome. The search was conducted using the following tools: Science Direct, Scopus, MDPI, Google Scholar, Research Gate and other sources. It focused on original papers, scientific articles, monographs, reports, conference proceedings and short communications. Search keywords included: hemp, plant growth-promoting bacteria, hemp microbiome, plant–microbiome interactions, cannabidiol, microbial diversity, industrial hemp, endophytes and cannabis microbiota. Two hundred and twelve scientific papers were identified, all in English. Seventy-five percent of these were published between 2020 and 2026. The publications were then assessed for their analyses of the impact of microorganisms on the plant and for studies using classical culture techniques and molecular techniques to determine the composition, quality and quantity of *Cannabis* spp. microbiota. The impact of microorganisms on *Cannabis* spp. was analyzed by assessing changes in morphometric parameters and secondary metabolite content.

**Figure 3:**
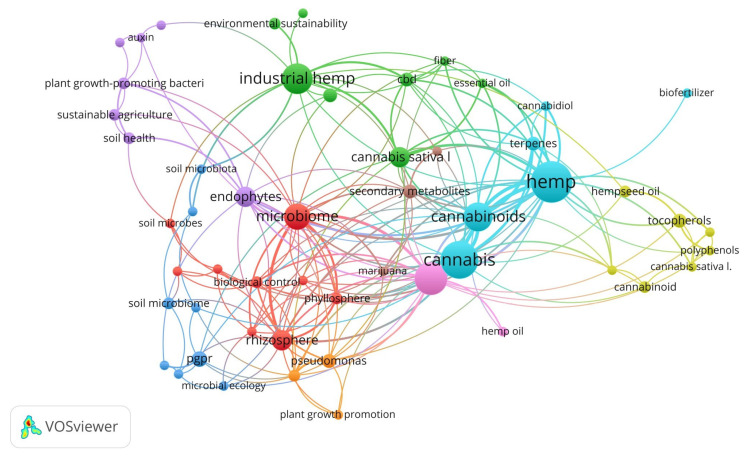
Network visualization of the publications used in the work. Colors represent different clusters of co-occurring keywords, indicating groups of closely related research topics identified using the VOSviewer clustering algorithm.

The publication set was analyzed using VOSviewer version 1.6.20, which grouped keywords by frequency. The minimum number of co-occurrences was set at 3. The size of the circles obtained from the analysis is proportional to the frequency of occurrence (Figure 3).

**Figure 4:**
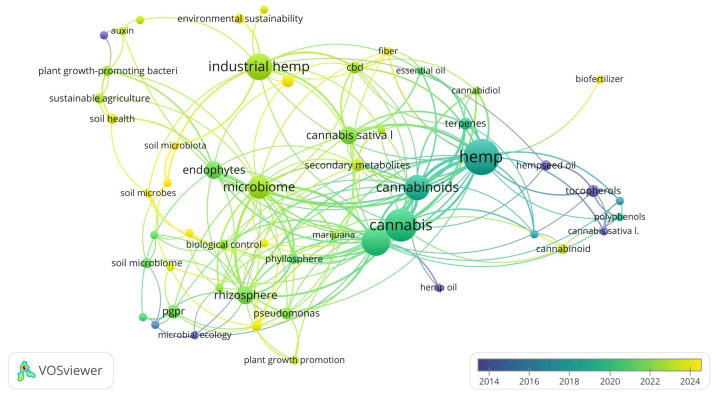
Web visualization of publications with the date of publication.

Based on the obtained map of keyword connections, a summary specifying the publication date was then created (Figure 4).

As a result of the analysis, 53 keywords were obtained, of which the most frequently occurring were hemp (25), *Cannabis* (22), *Cannabis sativa* (18), industrial hemp (16), cannabinoids (15), microbiome (13), rhizosphere (9), and endophytes (9).

## 4. Summary and Future Perspectives

Research conducted in recent years confirms that the composition of the cannabis microbiome is determined by both environmental factors and the plant’s genotype. The use of molecular methods significantly expands our knowledge of *Cannabis sativa* spp.-related microorganisms, indicating the presence of bacteria whose growth on media in laboratory conditions is impossible due to the network of interactions between microorganisms, specific culture conditions or slow growth.

Molecular analyses indicate the dominance of Proteobacteria, Actinobacteria, Firmicutes and Bacteroidota, while the share of individual microbial groups differs between industrial and medical varieties, and differences in composition also occur across tissues, indicating distinct colonization strategies and distinct roles.

Scientific reports also indicate plant–microorganism interactions, where cannabis of different genotypes can shape the microbial composition associated with the rhizosphere. Microorganisms, in turn, may influence the production of secondary metabolites in *Cannabis sativa* spp., but this effect is cultivar-specific and the mechanism remains to be further researched.

*Bacillus*, *Pseudomonas*, *Rhizobium*, *Burkholderia*, *Mucilaginibacter* and Serratia are also significant plant growth promoters in Cannabis. Their ability to increase biomass, improve germination, modulate the root system and exert antipathogenic effects has been noted. Multistrain inoculants, in many cases, demonstrated higher efficacy than single strains, confirming the complexity of interactions between microorganisms and the plant.

Future research should combine analysis of the cannabis genotype and chemotype with characterization of the microbiome and assessment of its biological functions. It is also important to determine the relationship between biodiversity levels and the functional stability of the agroecosystem. Furthermore, a combination of omics sciences should be employed to understand the impact of microorganisms on secondary metabolite biosynthesis pathways. Given the changes in agriculture, future research should also focus on developing effective growth-promoting formulations based on microorganisms as alternatives to chemical agents.

## Figures and Tables

**Table 1:** The effect of microorganisms on *Cannabis sativa* spp. shown in publications from 2020 to 2026. ↑ indicates a significant increase in the parameter value, ↓ indicates a significant decrease in the parameter value.

Country	Variety	Microorganism Tested	Place of Isolation	Effect on the Plant	Source
Canada (vase tests)	Fibrous variety: Anka	*Pseudomonas fluorescens* LBUM223; *Pseudomonas protegens* LBUM825; *Bacillus velezensis* LBUM279; *Bacillus subtilis* LBUM979; *Bacillus siamensis* LBUM1082	Own collection	↑ total plant dry matter	[56]
Greece (vase tests)	Fibrous variety: USO 31	*Rhizophagus irregularis*	Commercial product MycoPlant^®^ Polvo Grow	↑ root length ↑ dry shoot mass ↑ total dry matter	[57]
Greece (vase tests)	Fibrous varieties: Felina; Fedora 17	*Trichoderma harzianum*	Commercial product Trianum-P	↑ root density ↑ height and dry weight ↑ number and fresh weight of buds	[58]
Italy (vase tests)	Oil variety: Finola	*Azospirillum brasilense* ATCC 29710; *Gluconacetobacter diazotrophicus* ATCC 49037; *Herbaspirillum seropedicae* ATCC 35892; *Burkholderia ambifaria* PHP7	Own collection	↑ germination efficiency of plants infected with *Fusarium oxysporum* ↑ shoot and root length	[59]
Italy (in vitro tests)	Fibrous variety: Futura 75	*Sphingomonas* spp.	Endophytes of seeds and seedlings	↑polyphenol content ↑antioxidant activity	[60]
Thailand (vase tests)	Research variety: KKU05	*Rhizophagus prolifer* PC2-2 *Rhizophagus aggregatus* BM-3 g3	Own collection	↑ height of the above-ground part, leaf area, number of branches, number of inflorescences. ↑ dry weight of leaf, stem and inflorescence. ↑ root length, surface area and dry weight ↑ CBD and THC content	[61]
Mexico (in vitro tests)	Medical variety: Mango Elite Plus	*Bacillus* spp.	Hemp root zone isolates	↑ germination efficiency ↑ sprout root length	[62]
Canada (vase tests)	Medical variety: CBD Kush	*Bacillus* spp.; *Mucilaginibacter* spp.; *Pseudomonas* spp.	Own collection	↑ dry mass of inflorescences ↑ THC and CBD content ↑ total terpene content ↑ stem dry weight	[63]
Morocco (vase tests)	Medical varieties: Therapy; Euphoria; Critical; CBD Sweet and Sour Widow; CBD US	*Rhizophagus irregularis* *Trichoderma harzianum*; *Bacillus subtilis*; *Dictyosphaerium chlorelloides*; *Rhizopus irregularis* DAOM 197198	Commercial product Ferticann; own collection	↑ inflorescence biomass ↑ plant height ↑ content of selected phytocannabinoids in selected hemp varieties	[64]
Austria (field tests)	Fibrous variety: Eletta Campana	*Pseudomonas frigotolerans* C1141; *Serratia plymuthica* RR2-5–10; *Pseudomonas putida*; *Pseduomonas* spp.; *Bacillus* spp.	Seed endophytes	↑ germination efficiency ↑ development of secondary roots ↑ plant biomass, root length and seedling shoot length ↑ stem height and thickness	[65]
Thailand (vase tests)	Medical variety: Foi Thong Suranaree 1	*Bacillus velezensis* S141	Soybean rhizosphere	↑ dry mass of the plant, including stem, leaves, roots ↑ chlorophyll content	[66]
Mexico (aquaponic tests)	Hybrid variety: Belmont	*Pseduomonsa fluorescencs*, *Trichoderma harzianum*, *Trichoderma viride*, *Trichoderma reesei*	Commercial product Micorrizas	↑ length of the root and above-ground part ↑ root and aboveground biomass ↑ phenol and flavonoid content	[67]
Thailand (vase tests)	Medical variety: Foi Thong Suranaree 1	*Rhizobium dioscoreae*; *Pantoea dispersa*; *Paenibacillus azoreducens*; *Enterobacter cloacae*; *Acinetobacter johnsonii*; *Burkholderia cepacia*; *Pseudomonas* spp.	Endophytes of *Cannabis sativa* spp. (root, leaf, stem)	↑ root and shoot growth of the plant ↑ dry mass of shoot and plant ↑ height and dry weight of the plant.	[68]
Netherlands (aquaponic tests)	Medical varieties: Amnesia Haze; Gorilla glue	*Bacillus* spp.; *Pseudomonas* spp.; *Flavobacterium* spp.; *Burkholderia* spp.	Own collection	↑ CBGA content ↓ THC content	[69]

**Table 2:** Research on the microbiota of *Cannabis sativa* spp. using molecular methods from 2020 to 2026. OTU—operational taxonomic unit; bASV—bacterial amplicon sequence variant; ASV—amplicon sequence variant.

Country	Variety	Type of Analysis	Bacteria	Author
China	Industrial variety	Analysis of the microbiome composition at different stages of cannabis development	Alphaproteobacteria (20.64–35.44%); Acidobacteria_ Gp4 (6.81–8.57%); Actinobacteria (4.14–13.89%); Sphingobacteria (6.67–8.22%); Betaproteobacteria (5.62–6.32%); Gammaproteobacteria (4.80–6.37%); Cytophagia (3.18–4.38%); Gemmatimonadetes (3.07–3.61%)	[70]
Canada	Suver Hase diploid	Analysis of microbiome composition depending on ploidy	*Bacillus*; *Streptomyces*; *Klebsiella*; *Pseudomonas*; *Enterobacter*; *Aeromonas*; *Mycoplasma*; *Micromonospora*; *Mucilaginibacter*; *Ruficoccus*; *Staphylococcus*; *Burkholderia*; *Mycolicibacterium*; *Erwinia*; *Bilophila*; *Streptococcus*; *Agrobacterium*; *Alteromonas*; *Sphingomonas*; *Corynebacterium*; *Actinomycetes*; *Pantoea*; *Leclercia*	[71]
	Suver Haze triploid		*Bacillus*; *Enterobacter*; *Klebsiella*; *Agrobacterium*	
Austria	52 varieties from around the world	Analysis of seed endophyte composition using breeding and molecular methods	Gammaproteobacteria; Alphaproteobacteria; Bacilli; Actinobacteria; Bacteroidia	[72]
USA	Anka	Molecular analysis of microorganisms from selected plant tissues (leaf, root, flower) and soil microbiome core designation	8913 bacterial OTUs; Core: Comamonadaceae; *Massilia*; *Aquabacterium*; *Rhizobium*; *Pseudomonas*; *Sphingomonas*; *Methylobacterium*; *Hymenobacter*; *Microbacteriaceae*; *Bacillus cereus*; *Ralstonia*; *Bacillus*; *Bradyrhizobium*	[73]
China	Gansuqingshui Yunnan No. 1 Yunmaza No. 1 Huoma No. 1	Microbiome analysis, considering plant parts	Proteobacteria (67.26%); Cyanobacteria (14.40%); Firmicutes (8.07%); Actinobacteria (4.93%); Bacteroidetes (1.49%) Genus: *Rhizobium* (16.85%); *Pseudomonas* (3.14%), *Planococcusb* (1.99%), *Bacillus* (1.73%), *Sphingomonas* (1.34%)	[74]
Thailand	Foi Thong Suranaree 1	Analysis of the endophyte microbiome from selected parts of hemp plants, considering growth conditions: field and pot tests	Proteobacteria 85.18%; Firmicutes (7.41%); Actinobacteriota (3.7%); Myxococcota (3.7%)	[68]
Canada	Anka	Analysis of the impact of selected PGPB, considering changes in biodiversity	Proteobacteria; Actinobacteriota; Bacteroidota; Planctomycetota; Verrucomicrobia; Acidobacteriota	[56]
Canada	CBD Yummy CBD Shark Hash	Analysis of the microbiome composition by cannabis chemotype and development stage	Proteobacteria; Actinobacteria; Bacteroidetes; Verrucomicrobia; Chloroflexi; Planctomycetes; Acidobacteria; Armatimonadetes; WPS-2; Patescibacteria; FCPU426; Fibrobacteres; Gemmatimonadetess; Firmicutes; Dependentiae; Spirochaetes; Hydrogenedentes	[75]
Austria	40 varieties with varying degrees of domestication	Analysis of seed endophyte composition considering the degree of domestication	Core bacteria: Sphingomonas; Pelomonas; Ralstonia; Burkholderia; Bacillus; Staphylococcus; Pseudomonas; Enhydrobacter; Kosakonia	[65]
China	Industrial variety of *Cannabis sativa*	Analysis of soil microbial diversity in crops with different rotation systems over 3 years. Monoculture system and watermelon, potato and bean rotation	Proteobacteria 28.4–34.4%; Acidobacteria 19.4–23.2%; Bacteroidetes 10.3–12.9% Verrucomicrobia; Actinobacteria; Planctomycetes; Gemmatimonadetes; Chloroflexi; Candidatus Saccharibacteria; Firmicutes Nitrospirae; Cyanobacteria	[76]
USA	Tangerine	Analysis of soil and selected plant tissues to determine the microbiological composition	2170 bASVs root zone soil 342 bASVs leaves; 181 bASVs buds; 1141 bASVs root	[77]
Morocco	Medical Strains: Therapy; Euphoria, Critical; CBD Sweet and Sour Widow; CBD US	Analysis of the effect of microbial inoculation on the growth and production of secondary metabolites and the impact on the root zone microbiota.	5931 bacterial ASVs Candidatus Kaiserbacteria; Caulobacterales; Chthoniobacterales; Gemmatales; Planctomycetales; Rhizobiales; Saccharimonadales; Sphingomondales; Streptomycetales; Tepidispharales	[78]

## Data Availability

No new data were created or analyzed in this study.

## Abbreviations

The following abbreviations are used in this manuscript:

ACC	1-Aminocyclopropane-1-Carboxylate
APX	Ascorbate Peroxidase
ASV	Amplicon Sequence Variant
bASV	Bacterial Amplicon Sequence Variant
CAT	Catalase
CBD	Cannabidiol
CBDV	Cannabidivarin
CBG	Cannabigerol
CBGA	Cannabigerolic Acid
CFU	Colony Forming Unit
DNA	Deoxyribonucleic Acid
DREB	Dehydration-Responsive Element-Binding
IAA	Indole-3-Acetic Acid
IPT	Isopentenyltransferase
LEA	Late Embryogenesis Abundant
OTU	Operational Taxonomic Unit
PGPB	Plant Growth-Promoting Bacteria
RD29A	Responsive To Desiccation 29A
RNA	Ribonucleic Acid
SPAD	Soil-Plant Analysis Development
THC	Tetrahydrocannabinol
THCA	Tetrahydrocannabinolic Acid
